# Surgical outcomes in patients with small cell lung cancer: comparative analysis of computed tomograpy-detected patients with others

**DOI:** 10.1186/1477-7819-11-61

**Published:** 2013-03-08

**Authors:** Tomonobu Koizumi, Toshirou Fukushima, Kazutoshi Hamanaka, Takayuki Shiina, Kazuo Yoshida, Ryoichi Kondo, Ryouhei Yamamoto, Nobuhiro Nishizawa

**Affiliations:** 1 Comprehensive Cancer Therapy, Division of Clinical Oncology, Shinshu University School of Medicine, 3-1-1 Asahi Matsumoto Nagano, Matsumoto city 390-8621, Japan; 2 Respiratory Center Shinshu University School of Medicine, 3-1-1 Asahi, Matsumoto city, 390-8621, Japan; 3 Division of Thoracic Surgery, Chushin Matsumoto Hospital, 811 Kotobuki Toyooka, Matsumoto city, 399-0021, Japan; 4 Division of Thoracic Surgery, Saku Central Hospital, 197 Usuda, Saku, 384-0301, Japan

**Keywords:** Small cell lung cancer, Chemotherapy, Surgery, Survey, LD-SCLC, Early stage

## Abstract

**Background:**

It is shown that low-dose computed tomography (CT) screening is useful for a reduction in lung-cancer-specific mortality in heavy smokers. However, the information about effectiveness according to the histological types of lung cancer has not been adequately investigated especially small cell lung cancer (SCLC). The present study was performed to see the clinical benefit of CT screening in patients with SCLC following thoracotomy.

**Methods:**

We retrospectively reviewed the outcome in patients with early stage SCLC who initially underwent thoracotomy. The clinical stages and actuarial survival were estimated according to the three means of detection of SCLC: chest CT, radiographic screen, and symptomatically prompted cases.

**Results:**

Sixty-nine patients (men/women, 63/6; mean age, 70 years) with SCLC underwent thoracotomy between 1991 and 2010 including chest CT (*n* = 13), radiographic screening (*n* = 39), and symptomatically prompted cases (*n* = 17). Pathological staging information included stage IA (*n* = 25), IB (*n* = 8), IIA (*n* = 13), IIB (*n* = 5), IIIA (*n* = 11), and IIIB (*n* = 7). Median survival time was 30.0 (95% confidence interval (CI): 22.0 to 57.0) months, with overall survival at 5 years of 34.3% (95% CI, 23.47 to 47.3). Nine patients (69%) with stage I were detected by CT which was significantly higher than those in other detection arms. However, there were no significant differences in the survival between CT and other detection arms.

**Conclusions:**

CT examination may be useful for detection in early stage SCLC potentially suitable for surgery, but the contribution to better clinical outcome in patients with SCLC remains unclear.

## Background

Lung cancer is the leading cause of cancer mortality worldwide [1], including Japan [2]. Small cell lung cancer (SCLC) represents 10% to 15% of all lung cancers [3] and shows a high grade of malignancy with rapid growth and early widespread metastasis. As it is a virulent disease with high metastatic potential, it is usually considered a systemic disease at initial presentation. However, surgical resection has been commonly accepted in the management of the early stage of limited-disease (LD) SCLC [4]. Based on several institutional analyses [5-9] and population-based registration data [10-12], positive and favorable survival rates with initial surgery have been reported in selected patients with early stage SCLC.

On the other hand, the National Lung Screening Trial (NLST), a large randomized controlled trial designed to evaluate low-dose computed tomography (CT) screening for lung cancer in heavy smokers, demonstrated a decrease in lung-cancer-specific mortality in a CT screening population [13-15]. In addition, there have been many reports that CT can detect more of these lesions at an earlier stage than chest radiography [15,16]. We initially began a low-dose CT screening trial using a mobile CT unit in Japan [17-20], and CT screening for lung cancer has now been extended in Nagano Prefecture, Japan. We previously reported six cases with early stage SCLC detected by CT screening; four of these cases were successfully treated by initial surgical resection [18]. Thus, CT screening may influence the clinical outcome in patients with SCLC. Recently, Cuffe *et al*. [21] summarized 10 cases of SCLC identified in the CT screening and suggested the ineffectiveness of CT screening because of their identical survivals with those in general SCLC patients. However, there remains to be a scarcity of data focusing on screen-detected SCLC.

There were two objectives in our present study. First, we performed a retrospective review of SCLC patients who initially underwent surgical resection at three main hospitals in Nagano Prefecture and summarized the clinical characteristics. In addition, we divided patients according to the initial presentations or reasons for detecting diseases into the following three categories: chest CT, radiographic screen, and symptomatically prompted cases. Finally, we examined and analyzed the clinical stage and survival outcomes according to the three categories.

## Methods

Between January 1991 and December 2010, thoracotomy was performed in a total of 3,776 cases of primary lung cancer at Shinshu University (1,535 cases), Saku Central Hospital (1,576 cases), and Chushin Matsumoto Hospital (665 cases). Among them, 69 cases of SCLC (1.8%, of total thoracotomy for patients with primary lung cancer), who were initially treated for SCLC by pulmonary resection, were enrolled in the present study. There were 11 cases of SCLC treated by surgery after systemic chemotherapy, but these subjects were excluded from the analysis. The study subjects were identified and selected retrospectively from operation lists and the electronic clinical record search engine of hospitals. This study was conducted according to the principles of the Declaration of Helsinki. Patient privacy was protected when using individual information.

Clinical staging was evaluated by standard examination. All patients underwent physical examination, complete blood cell count, biochemistry examination, chest radiography (CXR), CT scans of the thorax and abdomen, bone scintigraphy, and magnetic resonance imaging (MRI) scan of the brain as pretreatment evaluation. Routine integrated positron emission tomography (PET)/CT scan was added to assess lymph node (and distant) involvement from 2005. Clinical and pathological staging were performed according to the 6th edition of the TNM classification of lung cancer. Routine mediastinoscopy was not performed in any cases. Operative indication was considered for clinical stages IA to IIB in patients with preoperative diagnosis of SCLC. With regard to diagnosis, routine fiberscopic bronchoscopy combined with transbronchial needle aspiration, forceps biopsy, brushing, and washing or percutaneous needle biopsy was performed if possible. However, when histological or cytological findings could not be obtained despite these examinations or when there was a strong suspicion of malignancy based on the CT findings, thoracotomy was recommended. In addition, when CT scans were indeterminate for lesions, such as those <10 mm, follow-up CT examinations were performed every 3 months to determine whether there were any interval changes in size, density, configuration, or internal structures. The interpretation of CT images was based on commonly used imaging features, that is, morphological characteristics and interval growing tendency [15,19,22]. Thus, when growing tendency in the nodules was definitely identified at the annual repeat CT screen, surgical resection was also recommended. In the absence of preoperative diagnosis, pathological examination was first determined intraoperatively, and in cases in which a diagnosis of malignancy was confirmed we proceeded immediately to extended resection of lung and lymph nodes. Systematically hilar and mediatinal lymph node dissection was performed in cases of lobectomy and pneumonectomy. Hilar and subcarinal lymph node (if necessary) sampling were done in case of partial resection. Final pathological diagnosis and stage were established based on the resected specimen and lymph nodes. Cases of large cell neuroendocrine carcinoma or mixed with non-SCLC were excluded from the present analysis.

The survival rates of the patients were calculated from the operation date until the time of death. Survival for all patients was recorded up to 31 July 2011. Operative mortality was defined as death within 30 days of operation or during the same hospitalization period.

### Statistical analysis

Statistical calculations were performed using StatView and SAS (SAS Institute, Inc., Cary, NC, USA). The actual overall survival rates after surgery were calculated using the Kaplan-Meier method, and differences in the resulting distributions were compared between groups by the log-rank test. Prognostic factors for overall survival were examined by the Cox proportional hazard model with adjustment for covariates, including sex, pathological stage, tumor size (>20 mm *vs*. ≤20 mm), lymph node metastasis status, post-chemotherapy (yes or no), type of resection, time period (1991 to 2000 *vs.* 2001 to 2010) and reason for admission (CT screen *vs*. CXR screen or symptomatic presentation). The numerical data in three groups were compared by analysis of variance with corrections for multiple comparisons (Scheffé’s test). Category data were analyzed using the chi-square test. The data are expressed as means ± standard deviation. In all analyses, *P* <0.05 was taken to indicate statistical significance.

## Results

### Clinical characteristics

The patient characteristics are shown in Table 1. The mean age was 70.0 years (range, 45 to 82 years). The study population consisted of 63 men (91.3%) and six women. Five of the six female patients were never smokers, but the others were smokers with a mean number of packs year of 62.1 ± 4.3. The pathological staging information included 25 patients with IA, eight with IB, 13 with IIA, five with IIB, 11 with IIIA, and seven with IIIB. Lobectomy was performed in 53 patients (76.8%), pneumonectomy was performed in three patients (5.0%), and segmentectomy or partial resection was performed in 13 patients (18.8%). Partial resection was done in elderly patients or cases with combined diseases such as chronic obstructive pulmonary disease, interstitial pneumonitis, or a history of prior thoracic surgery. Nine of 13 patients received partial resection were considered to be clinical stage IA and the stage was identical with the pathological stage in six patients. In preoperative evaluation, 40 cases were not confirmed histologically to be malignant. Preoperatively, there were nine and three patients with clinical stages IIIA and IIIB, respectively. Among the patients with IIIA, five patients were not confirmed to be malignant preoperatively, and four had suspected non-SCLC cytologically. For these patients, lobectomy and pneumonectomy were performed in eight patients and one patient, respectively. One patient with clinical stage IIIB had diagnosed as non-SCLC (squamous cell carcinoma) and performed pneumonectomy. However, other two patients with clinical stage IIIB, were not confirmed to be malignant preoperatively, and partial resection for the diagnosis was performed in the remaining two patients.

**Table 1:** Patient characteristics (total of 69 cases)

**Mean age**	**70.0 (45 to 82 years)**	
M : F	63 : 6	
Smoking history		
Never smoker	5	
Smokers	64	
	(Mean pack year 62.1 ± 4.3)	
Stage	Clinical	Pathological
IA	36 (52.2%)	25(36.3%)
IB	12 (17.4%)	8 (11.6%)
IIA	6 (8.7%)	13 (18.8%)
IIB	4 (5.8%)	5 (7.2%)
IIIA	8 (11.6%)	11 (15.9%)
IIIB	3 (4.3%)	7 (10.1%)
Surgery type		
Lobectomy	53 (76.8%)	
Partial resection	13(18.8%)	
Pneumonectomy	3 (4.3%)	
Post chemotherapy		
Yes	41 (59.4%)	
No	28 (40.6%)	

Postoperatively, 41 patients were treated with chemotherapy. By 1998, alternating cyclophosphamide/doxorubicin/vincristine with cisplatin/etoposide chemotherapy was performed. Subsequently, cisplatin/etoposide or cisplatin/CPT-11 was performed after resection. Chemotherapy was repeated for two to four cycles. There were no patients treated with adjustment thoracic radiotherapy for hilar-mediastinal area after surgery. The postoperative period was uncomplicated and there were no cases of postoperative in-hospital mortality. However, one patient developed pneumonia and died 2 months after surgery.

### Differences between clinical and pathological stages

We evaluated the relationships between clinical and pathological stages in 69 cases and the data are summarized in Table 1. Only two patients were overestimated in T- (clinical IB) and N-factor (clinical IIA) in patients with pathological IA stage, respectively. However, the clinical stage in 21 patients was underestimated; thus, the accuracy was 66.7%. The differences in N- and T-factors between clinical and pathological stages in the 21 patients are shown in Table 2. Thirteen of the 36 cases in clinical stage IA were underestimated. Differences in N-factor were observed in 16 patients, which was 23.2% of all subjects. The change in T-factor to T4 was due to pleural dissemination.

**Table 2:** Relationship between clinical and pathological stages in SCLC initially treated with thoracic surgery

		**Pathological stage**					
		**IA**	**IB**	**IIA**	**IIB**	**IIIA**	**IIIB**
**Clinical stage**	**IA**	23	2	8	1(T1→T2 N0→N1)	1	1
			(T1→T2)	(N0→N1)		(N0→N2)	(T1→T4)
	**IB**	1	6	1	1	3 (T2→T3,N0→N1)	
		(N1→N0)		(N0→N1)	(N0→N1)	(N0→N2)	
						(T2→T3,N0→N1)	
	**IIA**	1		4			1
		(N1→N0)					(T1→T4 N1→N2)
	**IIB**				3		1
							(T2→T4)
	**IIIA**					7	1
							(T2→T4)
	**IIIB**						3

Parentheses indicated the differences in N- and T-factors between clinical and pathological stages in mismatched patients (*n *= 23).

### Survival analysis

**Figure 1:**
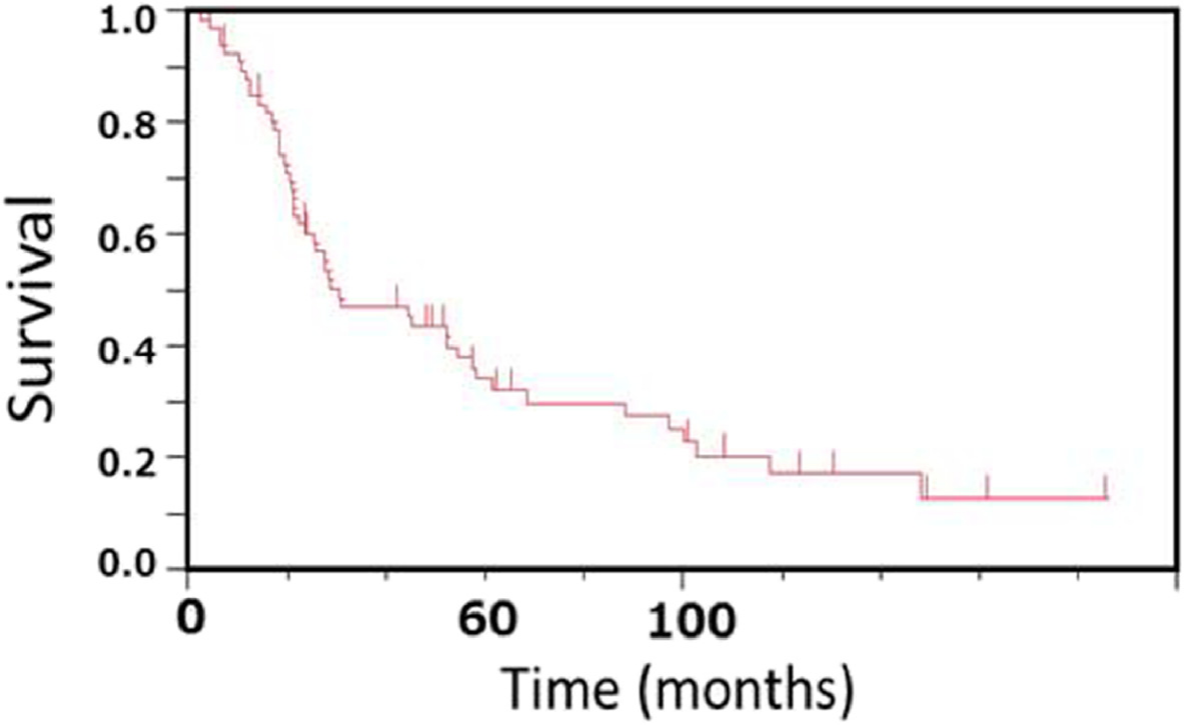
**Overall survival in patients with initially resected SCLC in the present study. **The median survival period was 30.0 (95% CI, 22.0 to 57.0) months with an overall survival (95% CI) at 5 years of 34.3% (95% CI, 23.47 to 47.3).

**Figure 2:**
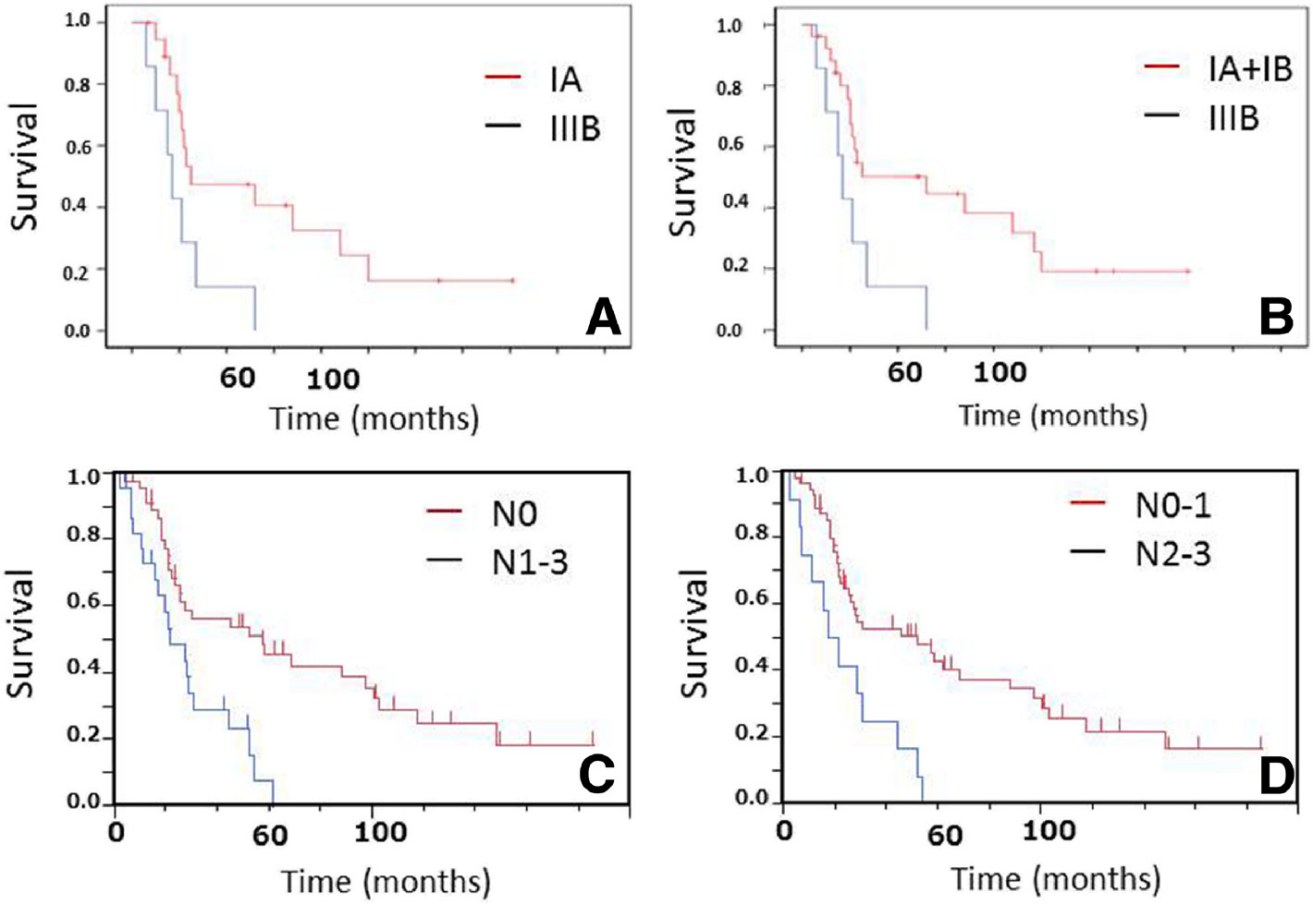
**Comparative analysis of overall survival according to the pathological stage revealed that the survival periods in patients stage IA and stage I (IA+IB) were significantly longer than that in stage IIIB (A, IA *vs. *IIIB, *P *<0.026; B, I *vs. *IIIB, *P *<0.019, respectively). **Comparative analysis of overall survival according to N-factors indicated that the survival in patients with N0 was significantly longer than that in N1-3 (**C**, *P *<0.0012). The survival in patients with N0-1 was also longer than that in N2-3 (**D**, *P *<0.0011).

**Figure 3:**
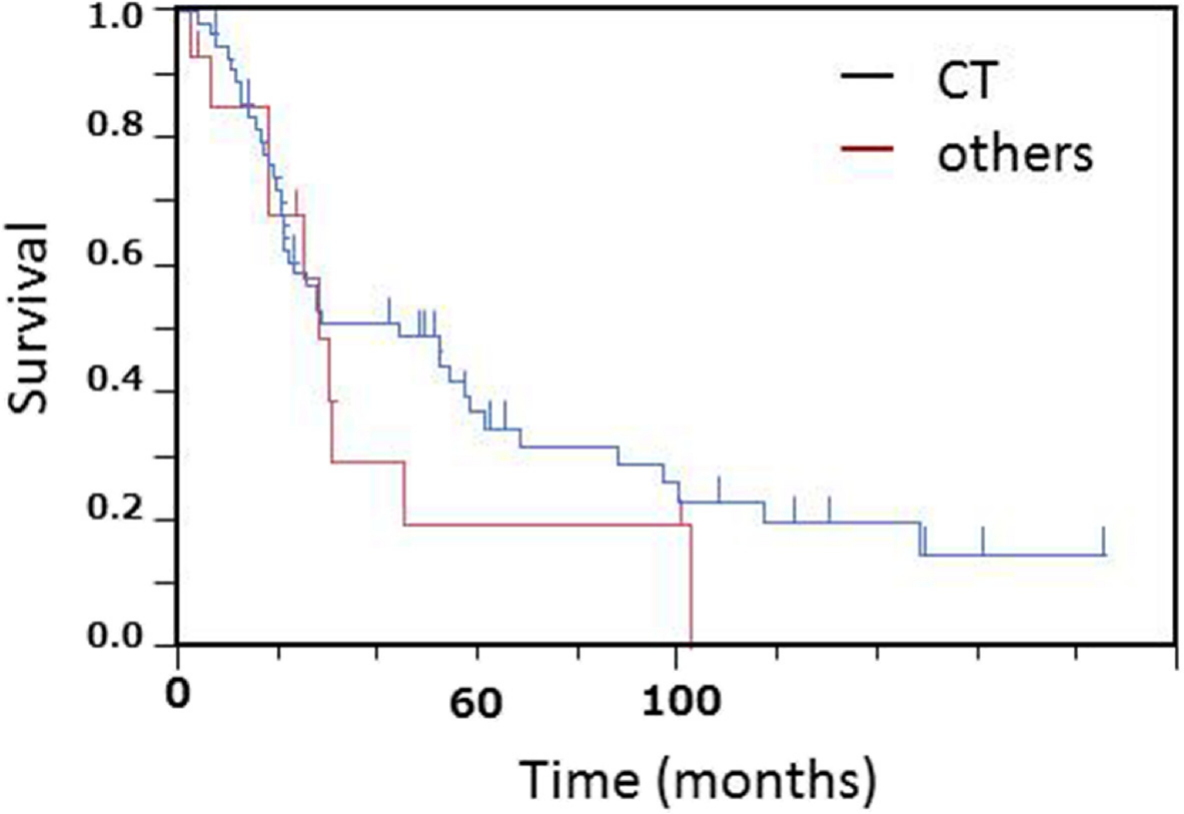
Comparative analysis of the overall survival between patients detected by computed tomography (CT) and others (chest radiographic screening test and symptomatic patients) showed no statistically significant difference between the two groups.

The overall survival curve is shown in Figure 1A. Median survival time and 5-year survival rate were 30.0 months (95% confidence interval (CI), 22.0 to 57.0) and 34.3% (95% CI, 23.47 to 47.3), respectively (Figure 1). According to the pathological stages in patients with resected SCLC, the median survival time and 5-year survival rate were 30 months (95% CI, 20.5 to 97.0) and 43.1% (95% CI, 26.5 to 61.5) in stage 1, 56.9 months (95% CI, 19.0 to 148.0) and 37.8% (95% CI, 17.6 to 63.4) in stage II, and 27.3 months (95% CI, 10.3 to 52.0) and 17.7% (95% CI, 5.8 to 42.8) in stage III, respectively. There were no statistically significant differences in overall survival among the three stages. However, survival periods in patients stages IA and I (IA+IB) were significantly longer than those in stage IIIB (IA *vs.* IIIB, *P* <0.026, Figure 2A, I *vs.* IIIB, <0.019, Figure 2B). In addition, in terms of N-factor, survival periods in patients with N0 and N0-1 were significantly longer than those in patients with N1-3 (N0 *vs.* N1-3, *P* <0.0012, Figure 2C) and N2-3 (N0-1 *vs.* N2-3, *P* <0.0011, Figure 2D), respectively. On the other hand, there were no significant differences between T1 and other T-factors (*P* <0.06). Furthermore, we divided tumor diameter into >20 mm *vs.* ≤20 mm, but there were no significant differences in survival between tumors of these two size ranges (*P* >0.50). Thus, the tumor size (T-factor) was not independent prognostic factor in patients with SCLC who were initially treated by thoracic surgery. We also analyzed the survival according to CT, CXR screen, and symptomatically prompted groups. Table 3 shows the number of patients in each pathological stage according to CT, CXR screen, and symptomatically prompted groups. Eight patients in the CT group were detected by CT screening. Five cases of SCLC were incidentally detected by chest CT due to the other diseases including postoperative follow-up for other malignant diseases or pulmonary diseases. There was no significant difference in the frequency and distribution of each stage between the CT group. Eight cases in CT group were stage IA, which was significantly higher than other groups. Survival curves in the CT group and other groups are shown in Figure 3. Survival in the CT screen group was better than in the chest CXR screen and symptomatically prompted groups, but the differences were not significant (*P* <0.37). Thus, there were no significant differences in survival among the three groups. In addition, there were no significant differences in survival related to sex, post-chemotherapy, type of resection, and time period between the first and later half (data not shown).

**Table 3:** Numbers of patients with SCLC initially treated with thoracic surgery according to CT test, radiographic screen (CXR), and symptomatically prompted cases

	**Symptoms (*n *=17)**	**CXR (*n *=39)**	**CT (*n *=13)**
Stage IA (*n *= 25)	4 (23.5%)	13 (33.3%)	8 (61.5%)^a^
Stage IB (*n *= 8)	1 (5.9%)	6 (15.4%)	1 (7.7%)
Stage IIA (*n *= 13)	3 (17.6%)	8 (20.5%)	2 (15.4%)
Stage IIB (*n *= 5)	1 (5.9%)	4 (10.3%)	0
Stage IIIA (*n *= 11)	3 (17.6%)	8 (15.4%)	0
Stage IIIB (*n *= 7)	5 (29.4%)	0	2 (15.4%)

^a^*Vs. *symptoms (*P *<0.05).

## Discussion

We summarized the clinical outcomes in patients initially underwent surgical resection for SCLC and demonstrated a 5-year survival rate of 34.3%. Several recent retrospective analyses have described improved or favorable survival rates in patient with resected SCLC [5-12]. Chandra *et al*. [5] summarized 67 patients, including four with stage IV disease, who underwent thoracotomy for SCLC at their own institution and described an overall 5-year survival rate of 27%. Lim *et al.*[9] recently described excellent results a 5-year survival rate of 52% for patients with stage I to III disease who underwent lung resection. In their series, there were no clear differences in clinical categories or across the spectrum of nodal disease from N0 to N2. In Japanese subjects with resected SCLC, 5-year survival rate was 43% and the survival has improved over the past several decades [8]. In addition, Schreiber *et al*. [11] summarized treatment arms and overall survival in patients with LD-SCLC using a US population-based database, and compared survival with and without surgery. They reported that 5-year survival rates in both T1-2, N0 and T3-4, N0 or T1-4, N1-2 were significantly higher in patients treated with than those in those without surgery (44.8% and 26.3% *vs.* 13.7% and 9.3%, respectively). Thus, surgery was associated with improved survival for selected patients with early stage SCLC. In our series, survival results and disease distribution were almost identical to these previous studies [5-12]. Thus, we also suggest that good results can be achieved in selected patients with complete resection throughout the spectrum of UICC from stage I to III. These results, including our data, do not suggest that we should routinely perform surgery in all patients in stages I to III. However, we feel that surgery could have an important role in the treatment strategies in patients in the early stages of LD-SCLC.

In the present study, N-factor rather than T-factor was an important prognostic factor in patients with SCLC who underwent initial thoracic surgery, which was consistent with previous studies [11,12,23]. Thus, an accurate preoperative diagnosis of the N-status should be made to determine indications for surgery in patients with SCLC. We found that the N-factor was underestimated in 16 patients in the present study. Similar findings of mismatch with nodal involvements between pre- and post-surgery were also reported in other studies [7,9,23]. Real-time endobronchial ultrasound-guided transbronchial needle aspiration (EBUS-TBNA) and PET/CT are new diagnostic tools and have been shown to be useful for precise diagnosis and staging of lung cancer, especially N involvements [24,25]. As EBUS-TBNA and PET/CT are now available for routine use in our institutes, the time frame of this 20-year retrospective analysis could influence the current data, which may be a limitation of the present analysis. Further studies are required to determine how N2 disease can be accurately diagnosed and excluded from surgery in patients with SCLC.

The present study focused on comparisons of staging and survival in initial resected SCLC according to the reasons for detecting disease, that is, CT and radiographic screening or symptoms. We found that a frequency of stage I SCLC in a CT detected population was significantly higher than those in other groups, but patients detected by CT had no better survival benefit compared with those detected by chest radiographic screening and symptomatically prompted patients. The prospective study of NLST demonstrated that the use of CT screening reduced mortality from lung cancer in heavy smokers [13-15]. However, CT screening identified a preponderance of early stage adenocarcinoma. Although survival analysis according to histological type was not described in the NLST study, there were no significant differences in detection of patients with SCLC in each stage between CT and radiographic screening groups [13]. The present study was limited to patients with initially resected SCLC and evaluated prospectively. Furthermore, CT group included five cases of SCLC accidentally detected by follow-up CT examination for other diseases. Thus, it is difficult for us to comment the role of CT screening on the prognosis in patients with SCLC from the present results. However, our data suggested that CT examination could bring a stage shift to early disease in SCLC who was considered for surgery. Further studies comparing the clinical outcomes of all stages of SCLC detected by radiographic screening with symptomatically prompted cases are required.

## Conclusions

Retrospective analysis of surgical results for SCLC suggested that operation should be considered for early stage SCLC. However, accurate preoperative diagnosis, especially N2 status, is required. In addition, CT screening may contribute to detection of early stage SCLC suitable for thoracic surgery but it remains unclear whether CT screening can improve clinical outcome in patients with SCLC.

## Competing interests

The authors declare that they have no competing interests.

## Authors’ contributions

TK participated in the design of the study and drafted the manuscript. TF participated in the data collection and analysis and helped to draft the manuscript. KH, TS, KY, RK, RY, and NN participated in the acquisition of each patient’s medical records and summarized the data in their own institutes. All authors read and approved the final manuscript.
